# Survival of opening versus closing wedge high tibial osteotomy: A meta-analysis

**DOI:** 10.1038/s41598-017-07856-8

**Published:** 2017-08-04

**Authors:** Jun-Ho Kim, Hyun-Jung Kim, Dae-Hee Lee

**Affiliations:** 1 Department of Orthopaedic Surgery, Samsung Medical Center, Sungkyunkwan University School of Medicine, Seoul, Korea; 2 Department of Preventive medicine, Korea University College of Medicine, Seoul, Korea

## Abstract

This meta-analysis was designed to compare the longevity of the survivorship of opening versus closing wedge high tibial osteotomy (HTO). All studies reporting survival rates in patients who underwent open or closed wedge HTO with more than 5-year follow-up duration were included in the meta-analysis. Survival time was considered as time to conversion to TKA. Twenty three studies were included in meta-analysis, 20 of which were of level IV evidence. The pooled 5-year survival rates were 95.1% **(**95% CI: 93.1 to 97.1%) in open wedge HTO and 93.9% (95% CI: 93.1 to 94.6%) in closed wedge HTO. Although there was 1.2% greater survival rate in open wedge HTO than in closed wedge HTO, this difference did not reach statistical significance (*P* = 0.419). Pooled 10-year survival rates were 91.6% **(**95% CI: 88.5 to 94.8%) in open wedge HTO and 85.4% (95% CI: 84.0 to 86.7%) in closed wedge HTO, indicating that open wedge HTO had 6.2% greater survival rate 10 years after surgery than did closed wedge HTO (*P* = 0.002). No difference in 5-year survivorship was found between open- and closed-wedge HTO. However, the survival rate was higher in open-wedge HTOs than in closed wedge HTO at 10 years.

## Introduction

High tibial osteotomy (HTO) has long been considered a successful and effective treatment option for relatively young and active patients with knee medial compartment osteoarthritis; HTO shifts the weight bearing axis to the relatively unaffected lateral compartment^1–5^. Two basic HTO techniques are commonly performed, a lateral closing-wedge HTO and a medial opening-wedge HTO^6, 7^. Traditionally, although closed-wedge HTOs were more common in the past^2^, the open-wedge HTO has gradually taken the place of the closed-wedge HTO^8^. Open-wedge HTOs have several advantages over closed-wedge HTOs, including easier control of the degree of correction, less extensive soft tissue dissection, sparing of the proximal tibiofibular joint, and the avoidance of serious complications such as peroneal palsy^2, 4, 9^. Previous studies have concentrated on comparing the two techniques with regard to correction angle, posterior tibial slope, patellar height, and complications. These findings not only correlated with postoperative outcomes but also provided important information to assist surgeons in choosing the appropriate treatment method^4, 6, 10^. However, these comparative studies have not consistently demonstrated either technique to be superior to the other. Surgeons choose between the two techniques based on personal preference, a discrepancy in the lengths of the patient’s legs, and/or a biomechanical abnormality such as ligament laxity of the knee^10, 11^. Given that the primary goal of HTO is to delay the time to total knee arthroplasty (TKA), it is important for both patients and surgeons to know whether medial opening or lateral closing wedge HTOs have the longest survival. Up to now, to the best of our knowledge, there have been only three meta-analyses that compared the clinic-radiological outcomes between medial opening and closing wedge HTOs^6, 12^, but no meta-analysis has evaluated the survival rate between these two methods. In addition, there is no general consensus on the approximate longevity of survivorship from the midterm to the long-term period after opening- and closing-wedge HTO^4^.

Therefore, this meta-analysis was designed to compare the longevity of the survivorship of opening- and closing-wedge HTOs and to quantify the approximate survival rates of both techniques. This study hypothesized that the survival rate would be different between opening- and closing-wedge HTOs, and that the survival rate of opening-wedge HTOs would be slightly higher than that of closing-wedge HTOs at the long term follow-up.

## Results

### Identification of studies

**Figure 1:**
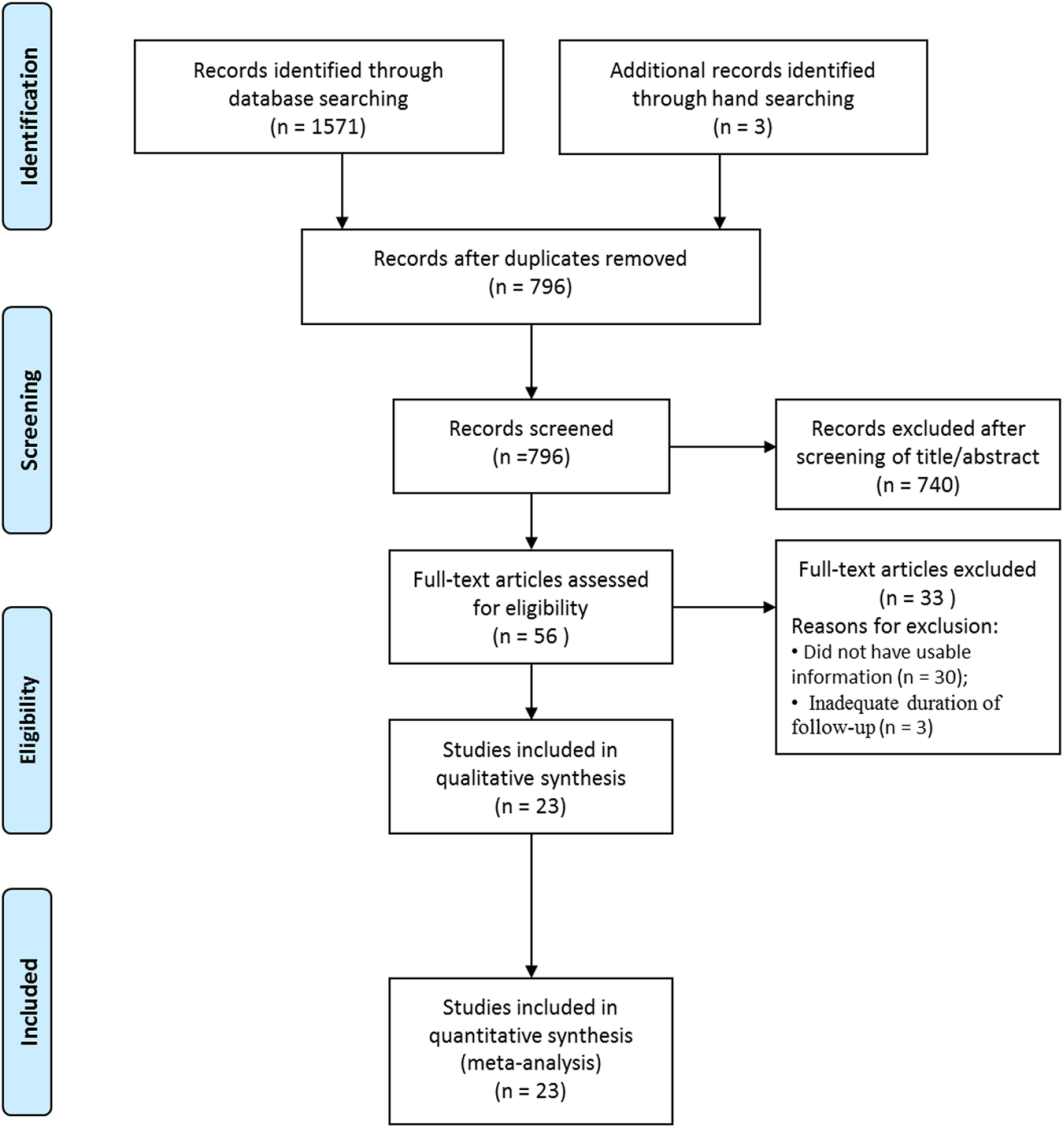
PRISMA (Preferred Reporting Items for Systematic reviews and Meta-analyses) flow diagram of the identification and selection of the studies included in this meta-analysis.

Figure 1 shows details of study identification, inclusion, and exclusion. An electronic search yielded 341 studies in PubMed (MEDLINE), 383 in EMBASE, 432 in Web of Science, 386 in SCOPUS, and 29 in the Cochrane Library. Three additional publications were identified through a manual search. After 778 duplicates were removed, 796 studies remained. Of these, 740 were excluded as it was clear from their abstracts and titles that they did not fulfill the selection criteria. An additional 33 studies were excluded because they did not provide usable information regarding survival rate or did not reach the adequate follow-up duration. Thus, 23 studies^7, 13–33^ were finally included in this meta-analysis.

### Study characteristics and patient populations

Of the 23 studies included in the meta-analysis, 3 reported the survival rate both in open and closed wedge HTOs. Sixteen studies reported the survival rate of closed wedge HTOs, with four reporting the survival rate of open wedge HTOs. Among 23 included studies, 20 studies were observational case series either in open or closed wedge HTOs. Two studies retrospectively compared the survival rate between open and closed HTOs, and only one study compared the survival rate prospectively between open and closed HTOs. In terms of the duration of the survival rate, two studies reported 5-year survival rates, four studies reported 10-year survival rates, and 17 studies reported 5- and 10-year survival rates simultaneously (Table 1).

**Table 1:** Summary of study characteristics.

Study	Year	Study type	Sample size, n		Quality score	Survival, year
			OWHTO	CWHTO		
Akizuki *et al*.^13^	2008	OCS		159	5	5, 10
Bae *et al*.^14^	2016	OCS		150	6	5, 10
Billings *et al*.^15^	2000	OCS		69	8	5, 10
Duivenvoorden *et al*.^16^	2015	RCS	112	112	6	5, 10
Efe *et al*.^17^	2011	OCS		199	7	5, 10
Flecher *et al*.^18^	2006	OCS		372	4	5, 10
Giuseffi *et al*.^19^	2015	OCS	89		6	5
Hernigou and Ma^20^	2001	OCS	245		8	5, 10
Howells *et al*.^21^	2014	OCS		95	5	5, 10
Hui *et al*.^22^	2010	OCS		413	6	5, 10
Koshino *et al*.^23^	2004	OCS		75	8	5, 10
Michaela *et al*.^24^	2008	OCS		134	6	5, 10
Naudie *et al*.^25^	1999	OCS		106	7	5, 10
Papachristou *et al*.^26^	2006	OCS		44	4	10
Schal. lberger *et al*.^7^	2011	RCS	56	16	6	5, 10
Schuster *et al*.^27^	2015	OCS	91		8	5
Sprenger and Doerzbacher^28^	2003	OCS		76	5	5, 10
Stukenborg-Colsman *et al*.^29^	2001	OCS		32	5	5, 10
Tang and Henderson^30^	2005	OCS		67	6	5, 10
van Raaij *et al*.^31^	2008	OCS		75	6	10
van Raaij et al.^32^	2009	OCS		77	8	10
van Egmond *et al*.^45^	2016	PCS	25	25	7	5, 10
Villatte *et al*.^33^	2015	OCS	69		4	10

CWHTO, closed-wedge high tibial osteotomy; OWHTO, open-wedge high tibial osteotomy; OCS, observational case series; RCS, retrospective comparison study; PCS, prospective comparison study

**Figure 2:**
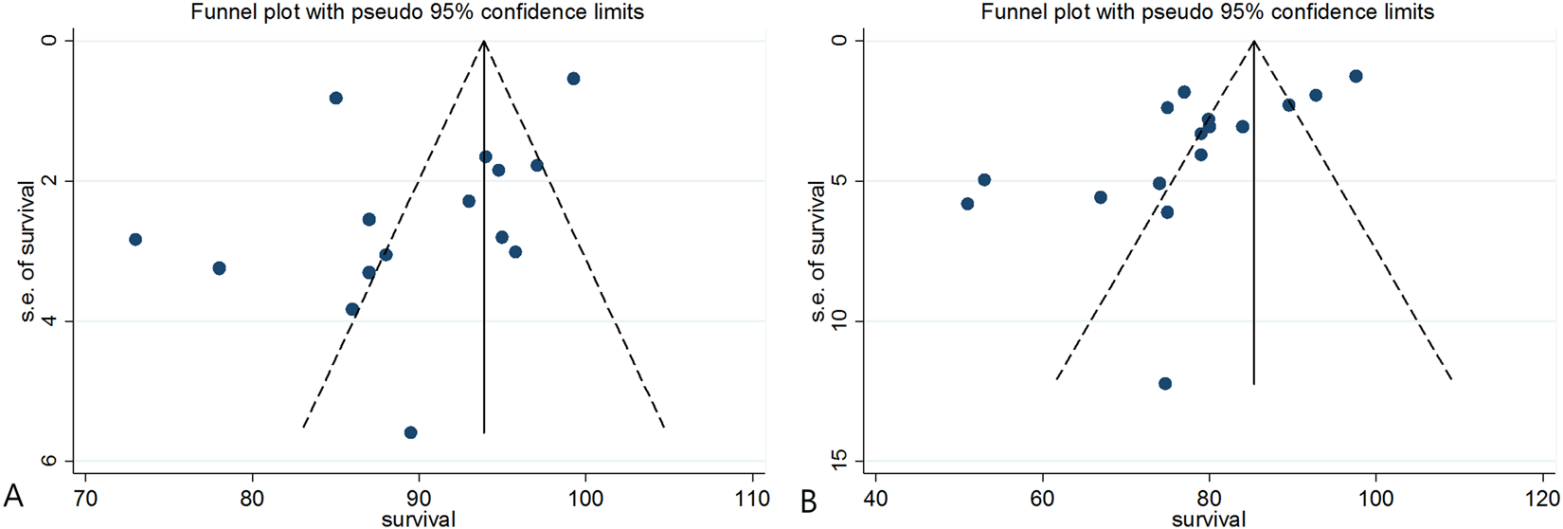
Funnel plot showing relatively symmetrical data on (**A**) 5-year survivalship of closed wedge high tibial osteotomy (HTO) and asymmetricity on (**B**) 10-year survivalship of closed wedge HTO.

All 23 studies included in this meta-analysis had a low risk of selection bias. None assessed possible confounding factors. Of these 23 studies, 16 were considered high quality, with > 5 points on the NOS. Inter-rater reliabilities (к values) for all items of the NOS ranged from 0.68 to 0.88, indicating at least a more than substantial agreement between the two investigators. In general, publication bias did not need to be evaluated if fewer than 10 studies were included. Therefore, we only assessed the publication bias of 5-and 10-year survival rates of closed wedge HTO. Funnel plots showed that the mean survival rate of closed wedge HTOs were relatively symmetric at 5 years (Fig. 2A), but skewed left asymmetrically at 10 years (Fig. 2B), indicating a lack of publication bias at the 5 year survival rate but some publication bias at the 10 year survival rate among the included studies. Egger’s test also confirmed these trends of publication biases, with no significant publication bias in survival rates at 5 years (*P* = 0.109), but some publication bias at 10 years (*P* = 0.012).

### Midterm survival rate

**Figure 3:**
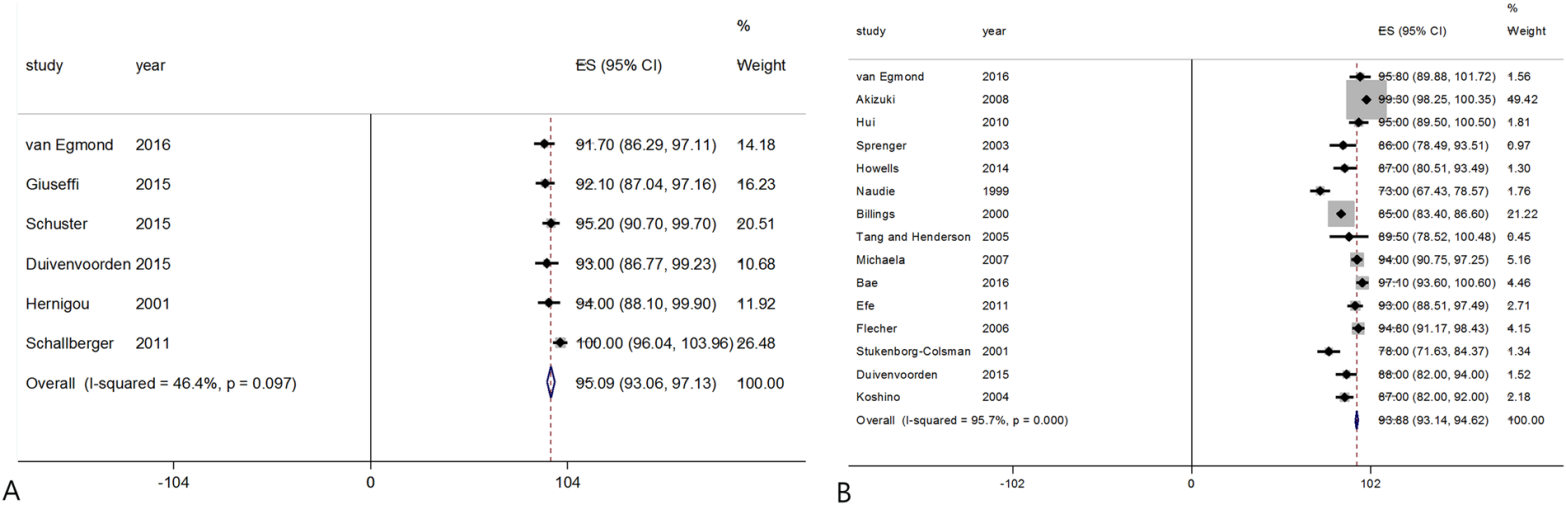
Forest plot showing the pooled 5-year survival rates of open wedge (**A**) and closed wedge (**B**) high tibial osteotomies.

Of the 23 studies, six included 578 knees that underwent medial opening wedge HTO and described the survival rate at 5 years after surgery, and 15 included 2084 knees that underwent lateral closing wedge osteotomy and described the survival rate at 5 years after surgery. The pooled 5-year survival rates were 95.1% **(**95% CI: 93.1 to 97.1%) in open wedge HTO and 93.9% (95% CI: 93.1 to 94.6%) in closed wedge HTO, respectively (Fig. 3A and B). However, although there was a 1.2% greater survival rate in open wedge HTOs than in closed wedge HTOs, this difference did not reach statistical significance (*P* = 0.419).

### Long-term survival rate

**Figure 4:**
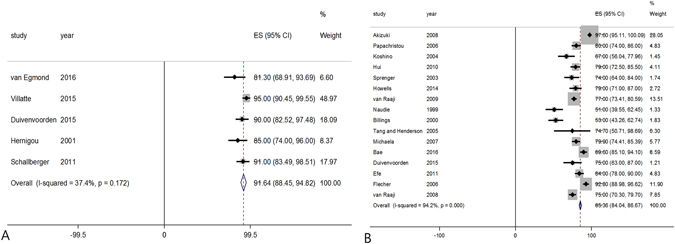
Forest plot showing the pooled 10-year survival rates of open wedge (**A**) and closed wedge (**B**) high tibial osteotomies.

Of the 23 studies, five included 467 knees that underwent medial opening wedge HTOs and described the survival rate at 10 years after surgery, and 16 included 2496 knees that underwent lateral closing wedge osteotomy and described the survival rate at 10 years after surgery. The pooled 10-year survival rates were 91.6% **(**95% CI: 88.5 to 94.8%) in open wedge HTOs and 85.4% (95% CI: 84.0 to 86.7%) in closed wedge HTOs, respectively, indicating that open wedge HTOs had 6.2% greater survival rate at 10 years after surgery than did closed wedge HTOs (*P* = 0.002, Fig. 4A and B).

## Discussion

This study estimated the approximate survival rates of open- and closed-wedge HTO by pooling the results of previous studies, most of which were cases series that did not compare two techniques directly. Although the methodological quality of the pooled studies was insufficient to adjust for possible confounders, the present study showed that the survival rate of open-wedge HTO was higher than that of closed-wedge HTO at 10 years.

This meta-analysis was undertaken to compare the survivorship of open- and closed-wedge HTO on the treatment of symptomatic medial knee osteoarthritis with varus leg alignment. Survival rates of HTO were not significantly different at 5 years follow-up. Over time, both techniques exhibited decreased survival rates and closed-wedge HTOs decreased more than open-wedge HTOs. There are several possible reasons for the superior survival rate of open-wedge HTOs at 10 years. First, the open-wedge HTO is thought to allow a more accurate correction than closed-wedge HTO because it allows fine-tuning of the desired correction in both coronal and sagittal planes^10, 34–36^. A higher degree of precision can theoretically result in better mechanical alignment and possibly superior survivorship^10, 36^. Smith *et al*.^12^ reported in a previous meta-analysis that there was a statistically significant difference in the mechanical axis with a more precise correction following open-wedge HTOs. Sun *et al*.^6^ recently performed a meta-analysis that showed that open-wedge HTOs have a higher accuracy than closed-wedge osteotomy in cases of overcorrection and undercorrection, even though there was no statistically significant difference in the postoperative mechanical axis. Second, the dynamics of knee alignment is a possible reason for the inferior result of closed-wedge HTO. The presence of a lateral tibial thrust and a high knee adductor moment are well known risk factors of HTO for survivorship. One of the main disadvantages of closed-wedge HTO is the extensive lateral approach that inevitably affects the proximal tibiofibular joint and lateral collateral ligament. For this reason, closed-wedge HTOs are thought to result in a higher adductor knee moment and a higher possibility of persistent lateral thrust than open-wedge HTO postoperatively. Naudie *et al*.^25^ reported that preoperative lateral tibial thrust is significantly correlated with the failure of HTOs in uni- and multi-variate analysis. Prodromos *et al*.^37^ studied gait analysis after HTO, and showed that patients with a low knee adductor moment had better clinical results. Also, a medial opening-wedge HTO is a well-established procedure for the correction of proximal tibial vara with medial compartment osteoarthritis (OA). Proximal tibial vara has been reported in over 85% of cases with medial compartment OA and varus malalignment of the limb resulting from OA may be attributable to the loss of cartilage and bony height of medial proximal tibia^38, 39^. From a biomechanical aspect, opening the depressed medial proximal tibia is thought be a more reasonable procedure in terms of correcting the deformed lesion than closing the intact lesion of the proximal tibia. Finally, the development of fixation devices for use in an open-wedge HTO is another possible reason for the higher survival rate. Traditionally, an open-wedge HTO is associated with complications including implant failure, lateral cortical fracture, and delayed union or nonunion^6, 34, 40–42^. Since angle-stable locking plates were introduced, implant related complications have been reduced markedly because of the HTO’s increased stability^32, 34, 40–44^.

Our meta-analysis shows that the survival rate with open- and closed-wedge HTO was 95.1% **(**95% CI: 93.1 to 97.1%) and 93.9% (95% CI: 93.1 to 94.6%) at 5 years, respectively, and 91.6% **(**95% CI: 88.5 to 94.8%) and 85.4% (95% CI: 84.0 to 86.7%) at 10 years, respectively. Our study revealed the only closed-wedge HTO survival rate at 15 years [74.8% (95% CI 72.5 to 77.2)] because studies of the 15 year survival rate with open-wedge HTO were limited. To our knowledge, no specific meta-analysis to date has quantified the survival rate of open- and closed-wedge HTO. The quantified survival rates found in the present study provide useful information not only for orthopedic surgeons but also for patients suffering from medial compartment OA of knee. If conservative treatment of medial compartment OA fails, surgical options include HTO, unicompartment arthroplasty (UKA), or TKA^21^. Therefore, these quantified results provide more information to orthopedic surgeons for choosing the appropriate treatment methods, although the final decision should be made after considering all factors. In addition, our study’s comparison assists surgeons in choosing between two different HTO methods, unless a patient has a clear indication for one method over another. Similarly, patients are most concerned about clinical improvement and procedure survival when choosing between the two different HTO methods. Although clinical outcomes of open- and closed-wedge HTO have been compared in a few studies, previous meta-analyses showed no differences in most studies^6, 12^. However, the present meta-analysis shows that open-wedge HTO has a longer survival rate than closed-wedge HTO at 10 years follow-up and provides a quantitative survival rate for both open- and closed-wedge HTOs. According to these results, patients who are candidates for HTO can get a clearer understanding of the consequences of open- and closed-wedge HTO with regard to survivorship. These results are important for surgeons and patients alike. Patients often demand a lucid explanation of the specific surgical procedure and have access to enormous amounts of information through the internet regarding any recommended surgery.

The current study has some limitations. First, differences in study designs are a limitation of this study. Most of the studies included in this meta-analysis were observational studies that were of variable methodological quality resulting in some inherent heterogeneity. Second, the number of studies regarding the survivorship of open-wedge HTOs is smaller than that of closed-wedge HTOs because closed-wedge HTOs were introduced earlier. If more studies report the survivorship of open-wedge HTOs in the future, it would help overcome this limitation. A third limitation is the heterogeneity of the fixation devices and wedge components used. This is a major limitation since biomechanical studies have demonstrated that any differences between open- and closed-wedge HTOs may be because of the nature of the osteotomy procedure and the fixation device used^12, 32, 34, 40–45^.

In conclusion, this is the first meta-analysis that shows no difference in the 5-year survivorship of open- and closed-wedge HTOs, and the survival rate was higher in open-wedge HTOs at 10 years. In addition, we estimated the long-term survival rate of both open- and closed wedge HTOs, providing useful information to surgeons and patients. However, the clinical evidence about long term survival rate at 10 years should be interpreted with caution, given our finding that there may be some publication bias in the 10 year survival rate among the included studies. Randomized control trials with a robust design need to be conducted to draw definitive conclusions regarding which of the two techniques yields superior long-term survival rates.

## Methods

### Data & literature sources

This study was based on the Cochrane Review Methods. Multiple comprehensive databases, including MEDLINE, EMBASE, Web of Science, SCOPUS, and the Cochrane Library (January 1, 1987 to June 30, 2016), were searched for studies that evaluated the survival rate in patients who underwent opening and/or closing wedge HTO. There were no restrictions on language or year of publication. Search terms used in the title, abstract, MeSH, and keywords fields included “Osteotomy” [tiab] or “Tibial” [tiab] or “High” [tiab] or “Open or Opening” [tiab], or “Closed or Closing” [tiab], and “Osteotomy” [MeSH] or “Survival” [tiab]. After the initial electronic search, relevant articles and their bibliographies were searched manually. Articles identified were assessed individually for inclusion.

### Study selection

Study inclusion was decided independently by two reviewers, based on predefined selection criteria. Titles and abstracts were read; if suitability could not be determined, the full article was evaluated. Studies were included in the meta-analysis if (1) they reported the survival rate in patients who underwent open or closed wedge HTO; (2) their follow-up duration was ≥ 5 years; (3) they considered the survival time of HTO as the time to conversion to TKA, a clear endpoint for HTO failure because avoiding knee arthroplasty is one of the main reasons to perform HTO; and (4) they fully reported the concrete numbers of subjects included in the final analysis as well as the number, not only the percentages, of patients not requiring conversion to TKA.

### Data extraction

Two investigators independently recorded data from each study using a predefined data extraction form. Any disagreement unresolved by discussion was resolved by consensus or by discussion with a third investigator.

Variables recorded included: (1) type of HTO (i.e., opening and/or closing wedge HTO and sample size; (2) numbers and percentages of surviving procedures without conversion to TKA at last follow-up; and (3) follow-up duration. Studies were excluded if (1) they dealt with a different type of high tibial osteotomy (i.e. dome osteotomy); and (2) their follow-up duration was < 5 years.

### Assessment of methodological quality

Two investigators independently assessed the methodological quality of each study using the Newcastle-Ottawa Scale (NOS), as recommended by the Cochrane Non-Randomized Studies Methods Working Group. In this analysis, the NOS star system, which awards stars depending on the level of bias, was adjusted to a scale that included only low (one star), high, and unclear bias. Each study was judged on three criteria: the selection of the study groups, the comparability of the groups, and the ascertainment of either the exposure or outcome of interest for case-control and cohort studies. Studies of high quality were defined as a score > 5 points. Disagreements in scores were resolved by discussion and consensus between the two reviewers.

### Statistical analysis

The main outcome of the meta-analysis was to compare the 5-year and 10-year procedure survival rates between opening- and closing-wedge HTOs, with continuous variables reported as the mean survival rate and the 95% confidence interval (CI). These values were analyzed with a random effects model. Interrater reliability in assessing methodological quality was evaluated by kappa (к), with values of ≤0.40, 0.41–0.60, 0.61–0.80, and 0.81–1.00 indicating no, moderate, substantial, and almost perfect agreement, respectively. Heterogeneity among the studies was determined by estimating the proportion of between-study inconsistencies due to actual differences between studies, rather than due to random error or chance, using theI^2^ statistic, with values of 25%, 50%, and 75% considered low, moderate, and high, respectively. All statistical analyses were performed using RevMan version 5.2 and Stata/MP 13.0. Publication bias was also assessed using funnel plots and Egger’s test.

## References

[1] Spahn G (2013). The impact of a high tibial valgus osteotomy and unicondylar medial arthroplasty on the treatment for knee osteoarthritis: a meta-analysis. Knee Surg. Sports Traumatol. Arthrosc..

[2] Preston S, Howard J, Naudie D, Somerville L, McAuley J (2014). Total knee arthroplasty after high tibial osteotomy: no differences between medial and lateral osteotomy approaches. Clin. Orthop. Relat. Res..

[3] Fu D (2013). Comparison of high tibial osteotomy and unicompartmental knee arthroplasty in the treatment of unicompartmental osteoarthritis: a meta-analysis. J. Arthroplasty.

[4] Nha KW, Kim HJ, Ahn HS, Lee DH (2016). Change in Posterior Tibial Slope After Open-Wedge and Closed-Wedge High Tibial Osteotomy: A Meta-analysis. Am. J. Sports Med..

[5] Kyung HS (2016). High Tibial Osteotomy for Medial Knee Osteoarthritis. Knee Surg Relat Res.

[6] Sun, H., Zhou, L., Li, F. & Duan, J. Comparison between Closing-Wedge and Opening-Wedge High Tibial Osteotomy in Patients with Medial Knee Osteoarthritis: A Systematic Review and Meta-analysis. *J. Knee Surg* (2016). 10.1055/s-0036-1584189 27218480

[7] Schallberger A, Jacobi M, Wahl P, Maestretti G, Jakob RP (2011). High tibial valgus osteotomy in unicompartmental medial osteoarthritis of the knee: a retrospective follow-up study over 13-21 years. Knee Surg. Sports Traumatol. Arthrosc..

[8] Seo SS (2016). Complications and Short-Term Outcomes of Medial Opening Wedge High Tibial Osteotomy Using a Locking Plate for Medial Osteoarthritis of the Knee. Knee Surg Relat Res.

[9] Han SB (2013). A “safe zone” in medial open-wedge high tibia osteotomy to prevent lateral cortex fracture. Knee Surg. Sports Traumatol. Arthrosc..

[10] Duivenvoorden T (2014). Comparison of closing-wedge and opening-wedge high tibial osteotomy for medial compartment osteoarthritis of the knee: a randomized controlled trial with a six-year follow-up. J. Bone Joint Surg. Am..

[11] Magnussen RA, Lustig S, Demey G, Neyret P, Servien E (2011). The effect of medial opening and lateral closing high tibial osteotomy on leg length. Am. J. Sports Med..

[12] Smith TO, Sexton D, Mitchell P, Hing CB (2011). Opening- or closing-wedged high tibial osteotomy: a meta-analysis of clinical and radiological outcomes. Knee.

[13] Akizuki S, Shibakawa A, Takizawa T, Yamazaki I, Horiuchi H (2008). The long-term outcome of high tibial osteotomy: a ten- to 20-year follow-up. J. Bone Joint Surg. Br..

[14] Bae DK, Song SJ, Kim KI, Hur D, Jeong HY (2016). Mid-term survival analysis of closed wedge high tibial osteotomy: A comparative study of computer-assisted and conventional techniques. Knee.

[15] Billings A, Scott DF, Camargo MP, Hofmann AA (2000). High tibial osteotomy with a calibrated osteotomy guide, rigid internal fixation, and early motion. Long-term follow-up. J. Bone Joint Surg. Am..

[16] Duivenvoorden, T. et al. Adverse events and survival after closing- and opening-wedge high tibial osteotomy: a comparative study of 412 patients. *Knee Surg. Sports Traumatol. Arthrosc* (2015). 10.1007/s00167-015-3644-2 PMC5332482 26026274

[17] Efe T (2011). Closing-wedge high tibial osteotomy: survival and risk factor analysis at long-term follow up. BMC Musculoskelet. Disord..

[18] Flecher X, Parratte S, Aubaniac JM, Argenson JN (2006). A 12-28-year followup study of closing wedge high tibial osteotomy. Clin. Orthop. Relat. Res..

[19] Giuseffi SA, Replogle WH, Shelton WR (2015). Opening-Wedge High Tibial Osteotomy: Review of 100 Consecutive Cases. Arthroscopy.

[20] Hernigou P, Ma W (2001). Open wedge tibial osteotomy with acrylic bone cement as bone substitute. Knee.

[21] Howells NR, Salmon L, Waller A, Scanelli J, Pinczewski LA (2014). The outcome at ten years of lateral closing-wedge high tibial osteotomy: determinants of survival and functional outcome. Bone Joint J.

[22] Hui C (2011). Long-term survival of high tibial osteotomy for medial compartment osteoarthritis of the knee. Am. J. Sports Med..

[23] Koshino T, Yoshida T, Ara Y, Saito I, Saito T (2004). Fifteen to twenty-eight years’ follow-up results of high tibial valgus osteotomy for osteoarthritic knee. Knee.

[24] Michaela G, Pedross F, Liebensteiner M, Bach C (2008). Long-term outcome after high tibial osteotomy. Arch. Orthop. Trauma Surg..

[25] Naudie, D., Bourne, R. B., Rorabeck, C. H. & Bourne, T. J. The Install Award. Survivorship of the high tibial valgus osteotomy. A 10- to −22-year followup study. *Clin. Orthop. Relat. Res*. 18–27 (1999). 10546594

[26] Papachristou G (2006). Deterioration of long-term results following high tibial osteotomy in patients under 60 years of age. Int. Orthop..

[27] Schuster P (2015). Open-Wedge High Tibial Osteotomy and Combined Abrasion/Microfracture in Severe Medial Osteoarthritis and Varus Malalignment: 5-Year Results and Arthroscopic Findings After 2 Years. Arthroscopy.

[28] Sprenger TR, Doerzbacher JF (2003). Tibial osteotomy for the treatment of varus gonarthrosis. Survival and failure analysis to twenty-two years. J. Bone Joint Surg. Am..

[29] Stukenborg-Colsman C, Wirth CJ, Lazovic D, Wefer A (2001). High tibial osteotomy versus unicompartmental joint replacement in unicompartmental knee joint osteoarthritis: 7-10-year follow-up prospective randomised study. Knee.

[30] Tang WC, Henderson IJ (2005). High tibial osteotomy: long term survival analysis and patients’ perspective. Knee.

[31] van Raaij T, Reijman M, Brouwer RW, Jakma TS, Verhaar JN (2008). Survival of closing-wedge high tibial osteotomy: good outcome in men with low-grade osteoarthritis after 10–16 years. Acta Orthop..

[32] van Raaij TM, Takacs I, Reijman M, Verhaar JA (2009). Varus inclination of the proximal tibia or the distal femur does not influence high tibial osteotomy outcome. Knee Surg. Sports Traumatol. Arthrosc..

[33] Villatte G (2015). Opening-wedge high tibial osteotomy with a secure bone allograft (Osteopure) and locked plate fixation: Retrospective clinical and radiological evaluation of 69 knees after 7.5years follow-up. Orthop. Traumatol. Surg. Res..

[34] Kolb W (2009). Opening-wedge high tibial osteotomy with a locked low-profile plate. J. Bone Joint Surg. Am..

[35] Magyar G, Ahl TL, Vibe P, Toksvig-Larsen S, Lindstrand A (1999). Open-wedge osteotomy by hemicallotasis or the closed-wedge technique for osteoarthritis of the knee. A randomised study of 50 operations. J. Bone Joint Surg. Br..

[36] DeMeo PJ, Johnson EM, Chiang PP, Flamm AM, Miller MC (2010). Midterm follow-up of opening-wedge high tibial osteotomy. Am. J. Sports Med..

[37] Prodromos CC, Andriacchi TP, Galante JO (1985). A relationship between gait and clinical changes following high tibial osteotomy. J. Bone Joint Surg. Am..

[38] Cooke TD, Pichora D, Siu D, Scudamore RA, Bryant JT (1989). Surgical implications of varus deformity of the knee with obliquity of joint surfaces. J. Bone Joint Surg. Br..

[39] Sharma L (2007). The role of varus and valgus alignment in knee osteoarthritis. Arthritis Rheum..

[40] Lee DH, Ryu KJ, Kim JH, Soung S, Shin S (2015). Fixator-assisted Technique Enables Less Invasive Plate Osteosynthesis in Medial Opening-wedge High Tibial Osteotomy: A Novel Technique. Clin. Orthop. Relat. Res..

[41] Brosset T, Pasquier G, Migaud H, Gougeon F (2011). Opening wedge high tibial osteotomy performed without filling the defect but with locking plate fixation (TomoFix) and early weight-bearing: prospective evaluation of bone union, precision and maintenance of correction in 51 cases. Orthop. Traumatol. Surg. Res..

[42] Cotic M (2015). A matched-pair comparison of two different locking plates for valgus-producing medial open-wedge high tibial osteotomy: peek-carbon composite plate versus titanium plate. Knee Surg. Sports Traumatol. Arthrosc..

[43] Bode G (2015). Prospective 5-year survival rate data following open-wedge valgus high tibial osteotomy. Knee Surg. Sports Traumatol. Arthrosc..

[44] Luites JW, Brinkman JM, Wymenga AB, van Heerwaarden RJ (2009). Fixation stability of opening- versus closing-wedge high tibial osteotomy: a randomised clinical trial using radiostereometry. J. Bone Joint Surg. Br..

[45] van Egmond N, van Grinsven S, van Loon CJ, Gaasbeek RD, van Kampen A (2016). Better clinical results after closed- compared to open-wedge high tibial osteotomy in patients with medial knee osteoarthritis and varus leg alignment. Knee Surg. Sports Traumatol. Arthrosc..

